# Family history of ADHD associates with stronger problem‐solving skills amongst 2‐ to 3‐year‐olds

**DOI:** 10.1002/jcv2.70009

**Published:** 2025-03-12

**Authors:** Alexandra Hendry, Emily J. H. Jones, Linn Andersson‐Konke, Mary Agyapong, Tessel Bazelmans, Jannath Begum‐Ali, Mutluhan Ersoy, Amy Goodwin, Greg Pasco, Terje Falck‐Ytter, Mark H. Johnson, Tony Charman, and the EASE and STAARS Teams

**Affiliations:** ^1^ Department of Experimental Psychology University of Oxford Oxford UK; ^2^ Faculty of Science Centre for Brain and Cognitive Development School of Psychological Sciences Birkbeck College University of London London UK; ^3^ Department of Psychology Uppsala University Uppsala Sweden; ^4^ Psychology Department Institute of Psychiatry Psychology & Neuroscience King's College London London UK; ^5^ Department of Psychology Kastamonu University Kastamonu Turkey; ^6^ Department of Forensic and Neurodevelopmental Sciences Institute of Psychiatry Psychology and Neuroscience King's College London UK; ^7^ Department of Women's and Children's Health Karolinska Institutet Center of Neurodevelopmental Disorders (KIND) Centre for Psychiatry Research Karolinska Institutet Stockholm Sweden; ^8^ Department of Psychology University of Cambridge Cambridgeshire UK

**Keywords:** ADHD, autism, endophenotype, executive functions, generativity, problem‐solving

## Abstract

**Background:**

Attention‐Deficit/Hyperactivity Disorder (ADHD) is linked to strengths in creative problem‐solving amongst school‐aged children and adults. In contrast, autism (which frequently co‐occurs with ADHD) is associated with lower generativity, and perseverative responses during problem‐solving. Little is known about how ADHD and autism traits—or broader heritable autism and ADHD phenotypes—associate with problem‐solving skills in early childhood.

**Methods:**

129 UK 2‐ and 3‐year‐olds (exploratory dataset) and 74 Swedish 3‐year‐olds (confirmatory dataset) with and without a family history (FH) of ADHD and autism, completed a problem‐solving task. Parents reported on their 3‐year‐olds’ ADHD and autism traits using the Child Behaviour Checklist and Social Responsiveness Scale‐2. FH group differences in problem‐solving performance were tested using ANOVA (exploratory dataset, FH‐autism and FH‐ADHD as fixed factors) and *t*‐test (confirmatory and combined datasets split by FH‐ADHD). Linear regressions of problem‐solving success on autism/ADHD traits were run in both samples.

**Results:**

Compared with peers with no FH‐ADHD, children with FH‐ADHD showed higher problem‐solving success at 2 (partial *ω* ^2^ = 0.106) and 3 years (partial *ω* ^2^ = 0.045) in the exploratory dataset. In the confirmatory dataset, a FH‐ADHD‐and‐autism group trended towards higher success scores compared with a no‐FH‐ADHD group (comprising FH‐autism‐only and no‐FH ADHD‐or‐autism sub‐groups) but scores were only significantly higher for children with FH‐ADHD‐and‐autism when compared with children with no FH‐ADHD‐*or‐autism* (*g*_*s*_ = 0.977). ADHD (but not autism) traits were positively associated with problem‐solving performance in the exploratory (*β* = 0.212, *p* = 0.031) and combined samples (*β* = 0.173, *p* = 0.024). Effects were a consistent direction and magnitude, but not significant, in the confirmatory sample alone (*β* = 0.201, *p* = 0.103).

**Conclusions:**

Considering a child's family history alongside their neurodivergent traits may help to identify their likely strengths, and how to access them: Children with ADHD traits and/or a family history of ADHD are likely to have an aptitude for generative problem‐solving when presented with highly motivating, ecologically valid challenges.

## Graphical abstract

In this study 129 UK 2‐ and 3‐year‐olds, and 74 Swedish 3‐year‐olds, with and without a family history of ADHD and autism, completed a problem‐solving task. Our results show that children with ADHD traits and/or a family history of ADHD are likely to have an aptitude for generative problem‐solving when presented with highly motivating, ecologically valid challenges.

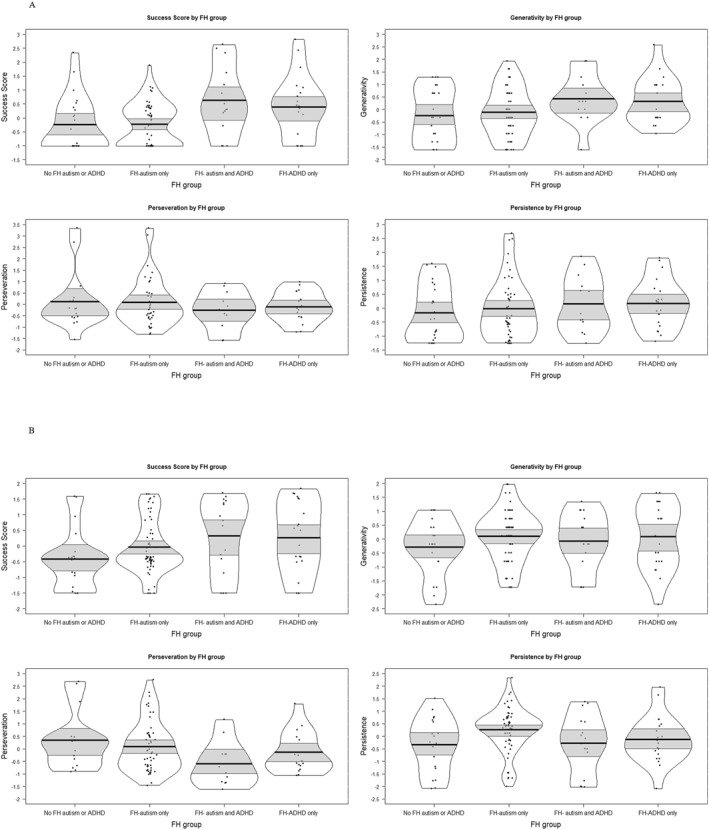

**Key points**

**What's known**

- Amongst children and adults, ADHD is linked to creative problem‐solving strengths, whilst autism is associated with lower generativity, and perseverative responses during problem‐solving.
- Little is known about how ADHD and autism traits associate with problem‐solving skills in early childhood.

**What's new**

- In two samples, we examine performance on a novel problem‐solving task amongst 2‐ and 3‐year‐olds with and without a family history of ADHD and/or autism, and associations with parent‐reported autism and ADHD traits.

**What's relevant**

- Young children with ADHD traits and/or a family history of ADHD tend to have an aptitude for generative problem‐solving when presented with highly motivating, ecologically valid challenges.

## ADHD‐RELATED STRENGTHS IN EARLY CHILDHOOD; AN UNDER‐EXPLORED AVENUE

Extensive research has documented cognitive tasks and processes which individuals with Attention‐Deficit/Hyperactivity Disorder (ADHD) tend to struggle with relative to non‐ADHD peers (Sonuga‐Barke et al., 2023). These difficulties include executive functions (cognitive processes that are involved in problem‐solving in order to attain a goal) such as inhibitory control and planning (Willcutt et al., 2005). Whilst understanding difficulties is necessary for identifying areas of potential support needs, it is also important to investigate areas of potential strength. This helps build a more accurate view of cognitive profiles associated with ADHD, and may reduce stigmatisation (thus potentially improving the wellbeing of those with ADHD) (Sonuga‐Barke & Thapar, 2021). Colzato et al. (2022) have argued that interventions that start from a strengths‐based approach are more effective than those embedded in a deficit model.

Prospective interventions for very young children afford the possibility of positively shifting developmental trajectories before difficulties become entrenched (Halperin et al., 2012). In such interventions, where children are recruited on the basis of elevated likelihood of ADHD but are not yet clinically diagnosed, there is a particular imperative to develop self‐confidence and build on strengths rather than focus solely on improving performance on cognitive tasks tapping into skills that may simply be developing on a non‐typical timeline. One candidate worth considering as an ADHD‐related strength that could be built upon is problem solving.

### Evidence for ADHD‐related advantages in problem‐solving

Previous work has indicated that ADHD may confer an advantage in certain types of problem‐solving and related cognitive tasks. ADHD has been linked to enhanced generativity or divergent thinking (i.e. the ability to come up with alternative solutions) (Girard‐Joyal & Gauthier, 2022; Gonzalez‐Carpio et al., 2017; Stolte et al., 2022; White & Shah, 2016), particularly on open‐ended tasks (Ten et al., 2020) or when competing for a bonus (Boot et al., 2020). Adults and adolescents with ADHD appear less constrained by prior knowledge during idea generation, relative to non‐ADHD peers (Abraham et al., 2006; White, 2020). These ADHD‐related strengths in generativity—which can be considered an aspect of executive function—can be contrasted with relative difficulties in problem‐solving tasks that require planning (another executive function component) and compliance with an arbitrary rule, such as Tower of London or Tower of Hanoi tasks (Patros et al., 2019).

### Identifying and recruiting young children with ADHD‐related traits

To date, participants in studies investigating ADHD‐related strengths in problem‐solving or generativity have been 6 years or older (Hoogman et al., 2020). One reason for this is that ADHD is not widely diagnosed before the age of 5 years (Davidovitch et al., 2017) due to difficulties in differentiating clinical traits from age‐appropriate (in)attentive and (hyper)active behaviours in very young children (Curchack‐Lichtin et al., 2014; Nili et al., 2023). Yet, several characteristics associated with the ADHD phenotype (e.g. surgency/approach behaviours, higher activity levels, lower inhibition, greater impulsivity) are at least partially evident in 0‐ to 5‐year‐olds with concurrent or later‐identified ADHD (Shephard et al., 2022). Further, activity levels and inhibitory control at age 2 years show predictive associations to ADHD traits at age 7 years (Shephard et al., 2019). Thus, with appropriate sampling, it should be possible to examine cognitive strengths in children showing ADHD‐related traits as early as toddlerhood, even if a reliable clinical diagnosis is not yet possible.

Finding a suitable sample in which to examine early ADHD‐related traits and cognitive strengths is feasible due to the high heritability of ADHD. Higher rates of ADHD are observed in parents and siblings of affected probands compared with relatives of unaffected controls (Biederman, 2005), with recurrence rates of ADHD amongst later‐born siblings of children with ADHD around 10%, in contrast to population‐prevalence rates of 3%–5% (Miller et al., 2019). Thus, recruiting infants or toddlers with a parent or sibling with ADHD (a ‘family history’ design) enables researchers to find children more likely to be on a pathway to ADHD, prior to any clinical diagnosis. Moreover, those children with a family history of ADHD who do not later meet clinical thresholds for ADHD are still more likely to show elevated levels of ADHD traits (Goodwin et al., 2021). This affords the possibility of also testing for cognitive advantages at subclinical levels of ADHD traits.

### Co‐occurrence of autism and ADHD

Complicating matters however, is the fact that ADHD often clusters with autism. Approximately 20% of children with ADHD score above clinical thresholds for autism (Ghirardi et al., 2018; Hollingdale et al., 2020). Further, individuals with a first‐degree relative with ADHD are more likely than average to be autistic, and vice‐versa (Ghirardi et al., 2018; Miller et al., 2019). Thus, samples recruited via an ADHD family history design are at elevated likelihood for autistic as well as ADHD traits. It is therefore important to consider whether cognitive traits observed amongst children with high ADHD traits and/or a family history of ADHD, are likely attributable to a heritable ADHD phenotype, a heritable autistic phenotype, or both.

### Evidence for autism‐related difficulties in problem‐solving

One way in which familial clustering and high co‐occurrence of ADHD and autism may influence problem‐solving skills is via cognitive flexibility. Cognitive flexibility is an important aspect of problem‐solving in general, and generativity or divergent thinking in particular (Hendry et al., 2022; Ionescu, 2012). Cognitive flexibility difficulties are not evident in all contexts for all autistic people (Leung & Zakzanis, 2014), and may be moderated by general intelligence and co‐occurring conditions (Kalbfleisch & Loughan, 2012; Kenworthy et al., 2008). Nevertheless, difficulties with cognitive flexibility are widely apparent amongst autistic pre‐schoolers (Christoforou et al., 2023). Regarding problem‐solving specifically, autistic children show lower generativity (Lai et al., 2017), and higher perseveration (Granader et al., 2014). Children with a family history of autism have been observed to experience some difficulties with cognitive flexibility, even if they do not meet diagnostic criteria for autism (Brunsdon & Happé, 2014; Rosa et al., 2017; Seng et al., 2020; St John et al., 2016; Van Eylen et al., 2017). When autism and ADHD co‐occur in the same individual this may give rise to a complex profile of strengths and difficulties relating to cognitive flexibility. For example, although children with co‐occurring autism and ADHD made more errors on a cognitive flexibility task and needed more time for the task compared with typically developing children, and those with autism only, children with co‐occurring autism and ADHD completed more stages (Sinzig et al., 2008).

### The current study

To date, no studies have investigated how a family history of ADHD interacts with a family history of autism with regards to early problem‐solving skills, nor how ADHD and autistic traits associate with problem‐solving skills prior to age 4 years. To address these questions, we present the results of an exploratory longitudinal study involving 129 children aged 2 and 3 years, attempting a problem‐solving task. We then try to replicate our exploratory findings in a separate sample of 74 3‐year‐olds, by testing the hypotheses that 1) having a family history of ADHD is associated with achieving higher success scores, 2) elevated ADHD traits are associated with higher success scores and 3) elevated autism traits are associated with higher success scores.

## METHOD

### Participants

#### Exploratory dataset

Participants were recruited in the UK if they had a first‐degree relative (parent or full sibling) with a family history of autism (FH‐autism), a first‐degree relative with ADHD (FH‐ADHD), or no first‐degree relatives with either autism or ADHD (No‐FH‐autism/ADHD). Each participant was coded for FH‐autism and FH‐ADHD status based on parent report of presence of autism and ADHD in family members (collected at study entry and confirmed/updated at the 2‐year visit, with follow‐up screening using age‐appropriate measures of clinical traits where ADHD was suspected; see Supporting Information S1: 1 for details). Of the 166 participants recruited for the overreaching study, 2 withdrew prior to the 24‐month assessment, 3 were excluded due to the first‐degree relative with a diagnosis being a half‐sibling not full‐sibling, and 32 did not contribute behavioural data at 24 or 36 months. These participants are not considered further, leaving 129 remaining.

As per previous studies from our group (Begum Ali et al., 2020) each participant was coded for family history of autism (FH‐autism; where ‘1’ indicates the presence of autism in a parent or sibling, ‘0’ indicates no known presence of autism in a parent or sibling) and family history of ADHD (FH‐ADHD; where ‘1’ indicates the presence of ADHD in a parent or sibling and ‘0’ indicates no known presence of ADHD in a parent or sibling). This approach allowed us to test the effect of FH‐autism, FH‐ADHD, and their interaction.

A rating of ‘FH‐autism = 1, FH‐ADHD = 1’ could apply where one family member had autism, and another family member had ADHD, or where one family member had both autism and ADHD. Although under versions of the Diagnostic and Statistical Manual of Mental Disorders prior to 2013 (APA, 2013) it was not possible to have a diagnosis of both autism and ADHD even if the individual showed strong traits of both, in the exploratory dataset (only) we did conduct follow‐up screening using age‐appropriate measures of clinical traits where ADHD was suspected but not formally diagnosed.

FH codes were used for analyses related to the research question, but additionally for descriptive purposes (e.g. Table 1) we computed a group variable whereby participants with a rating of ‘FH‐autism = 1, FH‐ADHD = 1’ were allocated to a FH‐autism+ADHD group.

**TABLE 1:** Characteristics of participants included in the final analyses, by FH group.

		No FH autism or ADHD	FH‐autism only	FH‐autism and ADHD	FH‐ADHD only	All
Exploratory dataset						
2‐year visit						
Age in months	Mean (SD)	24.06 (1.19)	25.43 (1.54)	24.87 (0.74)	25.22 (1.23)	25.25 (1.35)
	Min, Max	22.68, 27.75	23.84, 30.58	24.07, 26.96	24.10, 28.57	22.68, 30.58
Early learning composite	Mean (SD)	114.25 (17.91)	100.63 (20.76)	96.94 (17.12)	106.81 (21.73)	103.84 (20.59)
	Min, max	68, 140	53, 139	77, 132	70, 142	53, 142
Maternal education^a^	Mean (SD)	3.48 (0.67)	3.00 (0.71)	2.60 (0.74)	3.05 (0.80)	3.05 (0.76)
	Min, max	2, 4	1, 4	2, 4	2, 4	1, 4
N (*n* boys)		23 (12)	51 (25)	15 (10)	21 (13)	110 (60)
3 years visit						
Age in months	Mean (SD)	37.37 (1.86)	37.51 (1.24)	37.49 (1.48)	37.76 (2.73)	37.53 (1.72)
	Min, max	35.84, 43.89	36.07, 43.17	36.10, 41.13	36.03, 49.08	35.84, 49.08
Early learning composite	Mean (SD)	129.05 (11.75)	108.07 (18.65)	105.93 (19.90)	118.95 (19.47)	113.38 (19.65)
	Min, max	109, 146	75, 141	65, 131	66, 144	66, 146
Maternal education^a^	Mean (SD)	3.51 (0.61)	2.90 (0.72)	2.60 (0.74)	3.05 (0.80)	2.99 (0.76)
	Min, max	2, 4	1, 4	2, 4	2, 4	1, 4
*N* (*n* boys)		20 (12)	56 (27)	16 (11)	21 (12)	113 (62)
Confirmatory dataset						
Age in months	Mean (SD)	37.94 (3.30)	37.71 (2.53)	37.73 (2.89)	‐	37.76 (2.79)
	Min, max	36.13, 46.65	35.83, 47.44	35.90, 46.75	‐	35.83, 47.44
Early learning composite	Mean (SD)	124.1 (9.39)	98.1 (21.50)	93.6 (17.76)	‐	105.27 (16)
	Min, max	112, 147	49, 147	49, 127		49, 147
Maternal education^a^	Mean (SD)	3.87 (0.52)	3.31 (0.97)	3.22 (0.93)	‐	3.39 (0.90)
	Min, max	2, 4	1, 4	1, 4	‐	1, 4
*N* (*n* boys)		12 (7)	16 (15)	12 (15)		74 (37)

^a^ Highest maternal education level where 1 = Primary, 2 = Secondary, 3 = Undergraduate or equivalent, 4 = Postgraduate or equivalent.

#### Confirmatory dataset

Participants were recruited in Sweden if they either had a first‐degree relative with a family history of autism (‘FH‐autism’), or no first‐degree relative with autism (‘No FH‐autism’). Each participant was additionally coded for FH‐ADHD; where ‘FH‐ADHD = 1’ indicates the presence of ADHD in a parent or sibling and ‘FH‐ADHD = 0’ indicates no diagnosed presence of ADHD in a parent or sibling.

Of the 79 participants recruited for the study after the experimental task was introduced into the protocol, 2 were excluded as invalid due to problems with the video, and 3 did not attempt the problem‐solving task due to illness, distress prior to introduction of the task, or disinterest in the task. These participants are not considered further, leaving 74 remaining participants.

See Table 1 for included participants' age and developmental level (assessed with the Early Learning Composite (ELC) score of the Mullen Scales of Early Learning (Mullen, 1995)).

A one‐way ANOVA revealed no significant group differences in terms of age in the exploratory dataset (2‐year‐visit *p* = 0.416, 3‐year‐visit *p* = 0.908) or the confirmatory dataset (*p* = 0.963).

### Procedure

Data were collected at ages two (exploratory dataset only) and 3 years as part of the longitudinal BASIS and EASE studies. Informed written consent was provided by the participant's parent. Ethical approval was granted by the National Research Ethics Service (13/LO/0751) and the Research Ethics Committee, Department of Psychological Sciences, Birkbeck, University of London (exploratory dataset) and by the National Ethics Committee in Stockholm, Sweden (confirmatory dataset).

### Measures

#### Problem‐solving box task

**FIGURE 1:**
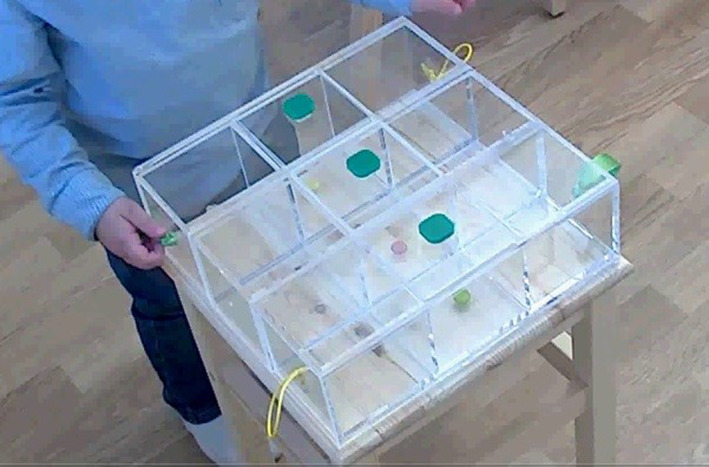
The Problem‐Solving box. To retrieve the central reward, the central green knob must be lifted. The retrieve the other rewards, the green ribbon must be pulled or the drawer pushed from the opposite face (i.e. the face with the yellow string).

The Problem‐Solving Box task (Hendry et al., 2022) is a reward‐retrieval task designed to mirror the kinds of problems that toddlers tackle in day‐to‐day life (namely, retrieval of a desirable object). The constraints of the task are embedded within the materials rather than requiring explanation or modelling of external rules: The Problem‐Solving Box is a transparent acrylic box with 3 compartments, each containing a small treat (e.g., chocolate/raisins); see Figure 1. The box is secured to a table 30 cm high. Each compartment has a green knob attached but only the central compartment lid can be lifted. The other 2 compartments are housed within sliding drawers. Tied through the hole at the end of each drawer is a string (which, if pulled, does not move the drawers) or ribbon (which, if pulled does move the drawers). Thus, to retrieve all 3 treats, participants must generate multiple strategies, inhibit previously successful or visually cued behaviours, and persist in the face of set‐backs. Success Scores show strong positive correlations with in‐task indices of generativity (where credit is given for any plausible goal‐directed strategy, whether or not it is successful), weak negative correlations with in‐task indices of perseveration on any one strategy, and moderate positive correlations with in‐task indices of persistence (Hendry et al., 2022). As generativity, inhibition and persistence can be characterised as executive functions, the Problem‐Solving Box may be considered an executive function task, and—since it involves extrinsic rewards that might be expected to be highly motivating or emotionally salient, it may more specifically be defined as a ‘hot’ executive function task (Hendry et al., 2016; Zelazo & Carlson, 2012). Of note, however, scores on measures of inhibitory control (widely considered a core executive function (Hendry et al., 2016; Miyake & Friedman, 2012) have been found to be inversely associated with performance on the problem‐solving box task (Hendry et al., 2022).

In the task warm‐up, the participant is given 1 treat and allowed time to eat it. In 7 cases (exploratory dataset FH‐autism‐only *n* = 4; FH‐autism + ADHD *n* = 2, confirmatory dataset *n* = 1) the participant was unwilling to eat the initial treat, so 3 small toys they had previously shown an interest in were used as rewards. The Problem‐Solving Box was then brought into view of the participant as the experimenter said ‘Here are more treats for you. Can you get them?’ The experimenter then moved to the side of the room. Once all 3 treats were retrieved or after 5 min, whichever was soonest, the experimenter terminated the task. If the participant did not touch the box after 1 min, the experimenter gave a prompt to continue. Further prompts were administered after approximately every 30 s of non‐touching. If the participant became distressed the task was terminated but to avoid biasing the data to represent only children who can persist in a moderately challenging 5‐min task, these participants' data were included if the task had been underway for more than 2 min (exploratory dataset *n* = 4). If any treats had not been retrieved at the end of the time limit, the experimenter demonstrated each action and observed whether or not the participant was able to retrieve after demonstration. If unable, this participant was excluded from analysis on the basis that the task was beyond their motor control abilities. Frequency and reasons for missing data are presented in Table S1.1.

Coders (trained to high inter‐rater reliability) rated participants' behaviour as described in Table S2.1. The primary variable was Success Score computed by subtracting the latency to achieve each reward from a total of 300 (maximum task duration, in seconds), then summing those values to produce a score ranging from 0 (unsuccessful problem‐solving) to 900 (successful problem‐solving).

Secondary variables (coded in the exploratory dataset only) were:

- Generativity; number of distinct goal‐directed strategies attempted. As detailed in Table S2.1, participants were given credit for a strategy even if did not directly lead to reward retrieval.
- Persistence; proportion of time spent on goal‐directed manipulation of the box
- Perseveration; duration of time spent on the dominant strategy (specific to each participant) as a proportion of the time spent on goal‐directed manipulation. So that this variable was not distorted by non‐engagement with the task, children spending less than 10 s on goal‐directed manipulation were excluded (2‐year visit *n* = 24, 3‐year visit, *n* = 14).

In many problem‐solving, creativity and executive function tasks, what should be considered a ‘good’ versus ‘bad’ outcome is subjective and linked to arbitrary rules. Toddlers in general, and neurodivergent children in particular, may have different objectives and perspectives on a task to a researcher (Hendry et al., 2016; Moreno‐Llanos et al., 2024; White, 2013). For example, in generativity tasks, researchers award higher scores the more novel solutions are listed, but the child may think it better to stop when they have identified what they consider to be the most appropriate or imaginative solution (Vaisarova & Carlson, 2021). In Tower‐type planning tasks participants score higher if they reach the (researcher‐defined) end‐point in the fewest moves; but to a child the value of the game may be in moving the pieces around as much as possible. This potential clash in perspectives is also an issue for the Problem‐Solving Box outcome measures of generativity (is it preferable to produce lots of strategies, or just effective ones?), persistence (is it preferable to stick with a difficult task, or temporarily withdraw to self‐regulate?) and perseveration (is it preferable to move onto a different strategy if one isn't immediately successful, or keep returning to it?). One context where researcher's and toddler participants' goals are likely to align is when the task involves retrieving treats. Based on years of engaging with toddlers, and observing their responses to the Problem‐Solving Box task we would argue that our operationalisation of an optimum outcome as the maximum number of rewards retrieved, as quickly as possible, almost always matches the participants'. Thus, we consider the Success Score to be the most appropriate outcome measure for the task.

#### Measure of ADHD traits

Parents completed the Child Behaviour Checklist for ages 1½ to 5 (CBCL; Achenbach & Rescorla, 2000). The ADHD DSM‐oriented scale of the CBCL comprises 6 statements rated on a three‐point Likert scale that assess a child's inattentive and hyperactive behaviour over the past 2 months. Item scores are summed to produce a total score which can be converted to t‐scores.

#### Measure of autism traits

Parents completed the Social Responsiveness Scale 2‐Preschool Form (SRS‐2; Constantino (2012). The SRS‐2 comprises 65 items rated on a 4‐point Likert scale anchored by ‘not true’ and ‘almost always true’. Item scores are summed to produce a total score which can be converted to t‐scores.

### Analysis

The effects of family history on problem‐solving performance were tested in the exploratory dataset using Analysis of Variance (ANOVA), with FH‐autism and FH‐ADHD as fixed factors, with the interaction of FH‐autism and FH‐ADHD included as a model term. As some variables had missing data, to preserve power separate univariate ANOVAs were run for each variable, but with correction for multiple comparisons.

To examine whether the primary exploratory finding (i.e. FH‐ADHD advantage in Success Scores) replicated in the confirmatory dataset, we pre‐registered a one‐tailed independent sample *t*‐test with Success Score as the outcome variable, and FH‐ADHD as the predictor variable; see Supporting Information S1: 3. Because the confirmatory dataset did not recruit any children with FH‐ADHD‐only (i.e. no FH‐autism), the pre‐registered contrast was between a FH‐ADHD‐and‐autism group and a children with either FH‐autism‐only or no‐FH‐ADHD‐or‐autism. No additional covariates were included in the models.

The associations between 3‐year autism and ADHD traits with problem‐solving performance were tested using linear regression in the exploratory dataset. On the basis of our exploratory findings, we pre‐registered tests of the hypothesis that ADHD traits (hypothesis 2) and autism traits (hypothesis 3) would be positively associated with Success Score in the confirmatory dataset; see Supporting Information S1: 3. In a variation from the pre‐registration, associations to total raw scores on ADHD and autism trait measures are reported rather than t‐scores because t‐scores showed low variance, and residual errors were not normally distributed. Total scores were log‐transformed to approximate a normal distribution where necessary, which was all cases except for CBCL‐ADHD scores in the exploratory dataset. Results of the regression analyses using t‐scores are reported in Supporting Information S1: 4. No additional covariates were included in the model.

## RESULTS

Table 2 presents descriptive statistics of problem‐solving performance and clinical measures, and results of group comparisons for problem‐solving performance. Correlations between problem‐solving box variables are reported in Supporting Information S1: 5.

**TABLE 2:** Scores on the problem‐solving box task and clinical measures, by FH group.

*Exploratory dataset: 2 year visit*											
	Descriptive statistics, by FH group							Hypothesis tests			
		No‐FH‐autism‐or‐ADHD	FH‐autism only	FH‐ADHD‐with‐FH‐autism	FH‐ADHD only	All		Model	Effect of FH‐autism	Effect of FH‐ADHD	Interaction: FH‐autism with FH‐ADHD
Success score	Mean	157.56	160.36	335.76	287.59	207.46	*F* (3,106)	4.834	0.385	13.813	0.305
	SD	200.37	156.61	253.07	226.92	205.17	Sig (2‐tailed)	0.003	0.536	< 0.001^a^	0.582
	Min, max	0, 687.98	0, 594.58	0, 749.46	0, 785.82	0, 785.82	Partial η^2^	0.120	0.004	0.115	0.003
	n	23	51	15	21	110	Partial ω^2^	0.094	0	0.106	0
Generativity	Mean	4.21	4.64	6.31	6.00	5.00	*F* (3,103)	2.294	0.313	6.849	0.009
	SD	3.30	3.02	3.01	2.81	3.10	Sig (2‐tailed)	0.082	0.577	0.010^a^	0.925
	Min, max	0, 9	0, 11	0, 11	2, 13	0, 13	Partial η^2^	0.063	0.003	0.062	0.000
	n	24	50	13	20	107	Partial ω^2^	0.035	0	0.052	0
Perseveration	Mean	0.46	0.46	0.40	0.43	0.45	*F* (3,79)	0.474	0.125	1.346	0.069
	SD	0.21	0.18	0.14	0.11	0.17	Sig (2‐tailed)	0.701	0.725	0.249	0.794
	Min, max	0.19, 1.00	0.23, 1.00	0.18, 0.60	0.25, 0.61	0.18, 1.00	Partial η^2^	0.018	0.002	0.017	0.001
	n	16	38	11	18	83	Partial ω^2^	0	0	0.004	0
Persistence	Mean	0.18	0.21	0.23	0.24	0.21	*F* (3,103)	0.522	0.107	1.366	0.141
	SD	0.17	0.18	0.17	0.15	0.17	Sig (2‐tailed)	0.668	0.744	0.245	0.708
	Min, max	0, 0.47	0, 0.66	0, 0.52	0.01, 0.51	0.00, 0.66	Partial η^2^	0.0.015	0.001	0.013	0.001
	n	24	50	13	20	107	Partial ω^2^	0	0	0.004	0

Exploratory dataset: 3‐year visit											
		No‐FH‐autism‐or‐ADHD	FH‐autism only	FH‐ADHD‐with‐FH‐autism	FH‐ADHD only	All		Model	Effect of FH‐autism	Effect of FH‐ADHD	Interaction: FH‐autism with FH‐ADHD
Success score	Mean	275.24	370.64	461.19	446.85	380.74	F (3,109)	2.279	1.092	6.231	0.596
	SD	236.76	219.11	302.41	289.65	253.18	Sig (2‐tailed)	0.084	0.298	0.014^a^	0.442
	Min, max	0, 781.54	0, 801.06	0, 808.75	0, 846.67	0, 846.67	Partial η^2^	0.059	0.010	0.054	0.005
	n	20	56	16	21	113	Partial ω^2^	0.032	0.001	0.045	0
Generativity	Mean	6.65	7.93	7.37	7.90	7.62	F (3,109)	0.844	0.298	0.261	1.736
	SD	3.31	3.01	3.34	3.74	3.25	Sig (2‐tailed)	0.473	0.586	0.610	0.190
	Min, max	1, 11	2, 14	2, 12	0, 13	1, 14	Partial η^2^	0.023	0.003	0.002	0.016
	n	20	56	16	21	113	Partial ω^2^	0	0	0	0.007
Perseveration	Mean	0.51	0.47	0.35	0.43	0.45	F (3,93)	2.417	2.425	6.393	0.187
	SD	0.19	0.17	0.15	0.14	0.17	Sig (2‐tailed)	0.071	0.123	0.013^a^	0.667
	Min, max	0.30, 0.90	0.21, 0.92	0.18, 0.65	0.27, 0.76	0.18, 0.92	Partial η^2^	0.072	0.025	0.064	0.002
	n	16	52	12	17	97	Partial ω^2^	0.042	0.015	0.054	0
Persistence	Mean	0.19	0.25	0.19	0.21	0.22	F (3,109)	2.610	1.140	0.674	3.161
	SD	0.12	0.10	0.13	0.10	0.11	Sig (2‐tailed)	0.055	0.266	0.413	0.078
	Min, max	0, 0.39	0.01, 0.47	0.01, 0.37	0.00, 0.43	0, 0.47	Partial η^2^	0.067	0.010	0.006	0.028
	n	20	56	16	21	113	Partial ω^2^	0.040	0.001	0	0.019
CBCL DSM ADHD score (*t*‐scores in parentheses)	Mean (SD) raw total score	3.05 (2.16)	4.18 (3.23)	5.79 (3.93)	4.95 (3.61)	4.31 (3.28)	Not tested				
	Mean (SD) t‐score	51.10 (1.45)	53.84 (6.19)	57.50 (8.94)	55.40 (7.86)	54.06 (6.57)					
	Min, max raw total score	0, 6	0, 11	0, 12	0, 12	0, 12					
	n	21	55	14	20	110					
SRS total score (*t*‐scores in parentheses)	Mean (SD) raw total score	23.37 (9.46)	43.65 (32.78)	61.00 (48.52)	34.45 (24.83)	40.15 (32.22)					
	Mean (SD) t‐score	42.89 (3.73)	50.77 (12.56)	57.33 (18.68)	47.15 (9.52)	49.38 (12.37)					
	Min, max raw total score	10, 41 38, 50	7, 123 37, 81	9, 159 37, 95	10, 118 38, 79	7, 159					
	n	19	52	12	20	103					

Confirmatory dataset											
		No FH autism or ADHD	FH‐autism only	FH‐ADHD with FH‐autism	FH‐ADHD only	All				Effect of FH‐ADHD	
Success score	Mean	294.91	457.43	493.96	‐	437.28	*t* (70)	‐		−1.633	
	SD	179.05	252.67	203.66		230.79	Sig (1‐tailed)	‐		0.053	
	Min, max	0, 694	0, 785	114, 807	‐	0.807	*d*_*s*_			0.398	
CBCL DSM ADHD score (*t*‐scores in parentheses)	Mean (SD) raw total score	1.21 (1.25)	3.53 (2.78)	4.68 (3.33)	‐	0.48 (3.01)	Not tested				
	Mean (SD) t‐score	50.07 (0.27)	52.40 (4.72)	54.52 (6.49)		52.70 (5.20)					
	Min, max raw total score	0, 4	0, 11	0, 11	‐	0, 11					
	n	14	30	25	‐	69					
SRS total (*t*‐scores in parentheses)	Mean (SD) raw total score	23.07 (13.29)	38.71 (26.32)	43.63 (27.45)	‐	37.51 (25.63)					
	Mean (SD) t‐score	42.71 (5.04)	48.50 (10.14)	50.74 (10.48)		48.39 (9.85)					
	Min, max raw total score	7, 52	9, 125	11, 107	‐	7, 125					
	n	14	31	27	‐	72					

*Note*: Partial η^2^ values indicate the proportion of variance associated with membership of that specific group (e.g. FH‐ADHD) after controlling for other factors in the model (e.g. FH‐ASD and the interaction effect of FH‐ASD and FH‐ADHD). As eta‐squared is considered a biased estimate (Lakens, 2013b) we also present partial ω^2^ values—computed using Lakens (2013a) and set to 0 for negative values. Both values can be interpreted using the heuristics suggested by Cohen (1988) where 0.01 is small, 0.06 is medium and 0.14 is a large effect.

Abbreviation: FH, Family History.

^a^ Significant after Benjamani‐Hochberg correction for 4 family wise tests.

### Effects of family history of autism/ADHD on problem‐solving performance

**FIGURE 2:**
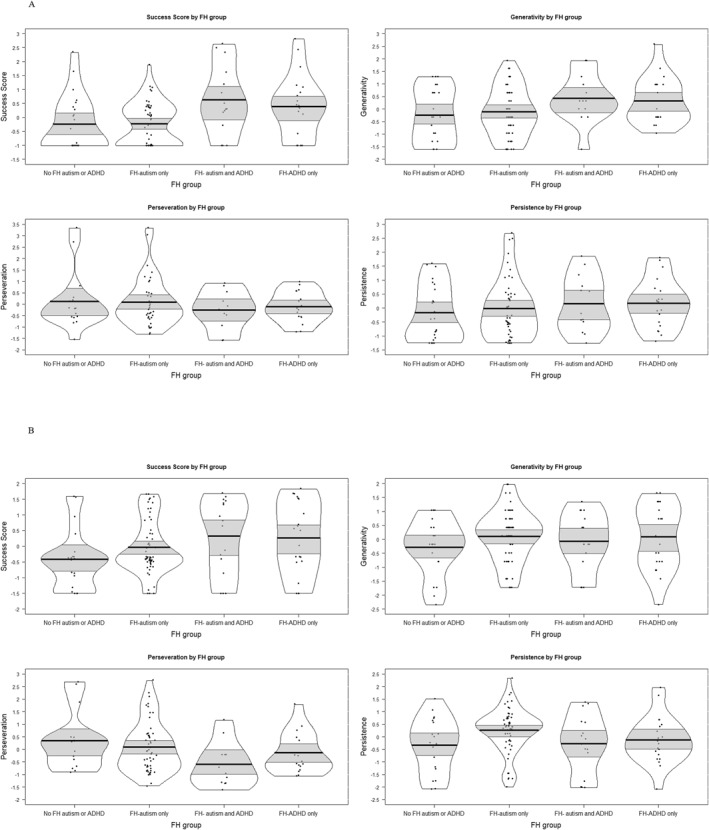
Standardised problem‐solving scores at (A) age 2 years, and (B) age 3 years by Family History (FH) group; exploratory dataset. The bold black line indicates the group mean, the light grey bands the 95% confidence interval, and the black border the full data distribution. Note that data is presented in FH subgroup for descriptive purposes only. Analyses were conducted using FH‐autism and FH‐ADHD codes as per Table 2.

As shown in Table 2, and Figure 2, in the exploratory dataset an FH‐ADHD advantage for problem‐solving was observed at both time‐points with regards to Success Score, with a medium‐to‐large effect size at age 2 years, and a small‐to‐medium effect size at age 3. Children with a FH‐ADHD showed higher generativity than their peers at age 2, and lower perseveration at 3 years, with a small‐to‐medium effect size in both cases.

**FIGURE 3:**
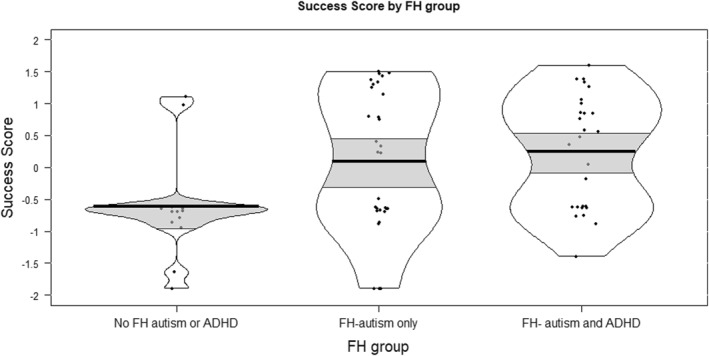
Standardised problem‐solving Success Scores at age 3 years, by Family History (FH) group; confirmatory dataset. The bold black line indicates the group mean, the light grey bands the 95% confidence interval, and the black border the full data distribution. Note that data is presented in FH subgroup for descriptive purposes only. Analyses were conducted using the FH‐ADHD code, as per Table 2.

In the confirmatory dataset (comparing a FH‐ADHD‐and‐autism group with children with either FH‐autism‐only or no‐FH‐ADHD‐or‐autism), the hypothesized FH‐ADHD advantage for problem‐solving did not reach significance (*p* = 0.053), but was in the predicted direction and of similar magnitude as the exploratory effect (see Table 2), with mean Success Scores for 3‐year‐olds with FH‐ADHD (and autism) of 494 (SD = 241), and mean Success Scores for 3‐year‐olds with No‐FH‐ADHD of 403 (SD = 204). As shown in Table 2 and Figure 3, in this dataset, the FH‐autism‐only group also showed elevated Success Scores relative to the No‐FH‐autism‐or‐ADHD group. Exploratory analysis in the confirmatory dataset found that 3‐year‐olds with FH‐ADHD‐and‐autism showed significantly higher Success Scores compared with just the No‐FH‐autism‐or‐ADHD group, with a large effect size (*t* (32.36) = −3.284, *p* = 0.001,*d*_*s*_ = 0.999, *g*_*s*_ = 0.977) (Equal variances not assumed: Levene's test for equality of variances *p* = 0.031).

Pre‐registered (after analysis of the exploratory dataset) *t*‐tests of the exploratory and confirmatory datasets combined confirmed an FH‐ADHD advantage for problem‐solving was observed with regards to overall Success Score at age 3 years, with a small‐to‐medium effect size (*t* (182) = −2.873, *p* = 0.002, *d*_*s*_ = 0.445, *g*_*s*_ = 0.443).

### Associations between problem‐solving box performance and traits of ADHD and autism

#### ADHD traits

**FIGURE 4:**
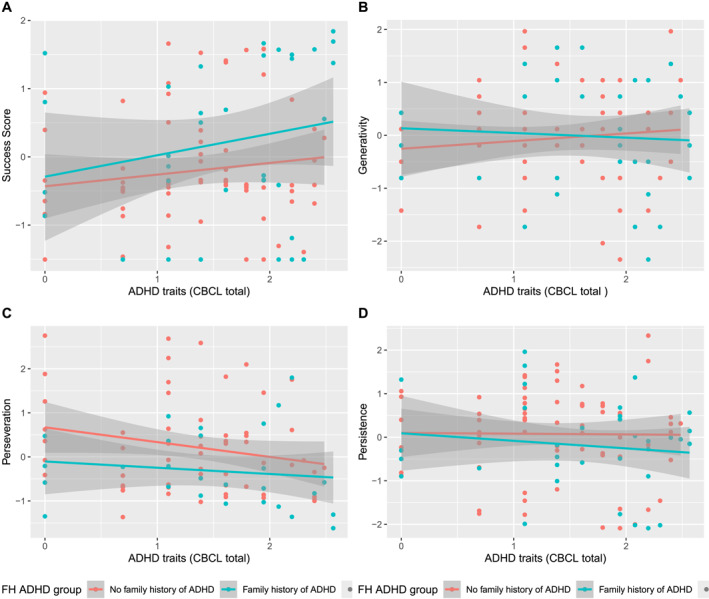
Regression of standardised problem‐solving variables (A) Success Score; (B) Generativity; (C) Perseveration; (D) Persistence) on ADHD traits (log transformed), by FH‐ADHD group in the exploratory dataset.

As shown in Table 3, in the exploratory dataset, at age 3 years ADHD traits were positively associated with Success Score, and negatively associated with Perseveration; see also Figure 4. In the confirmatory dataset, the ADHD trait‐Success Score association was of a similar magnitude and direction to the exploratory finding, but did not meet significance thresholds.

**TABLE 3:** Regression analysis of problem‐solving performance on autism and ADHD traits at age 3 years.

		Success score	Generativity	Perseveration	Persistence
Exploratory dataset					
CBCL‐ADHD total scores^a^	Beta (β)	0.212	0.016	−0.255	−0.099
	B, SE	0.003, 0.001	0.016, 0.099	−4.787, 1.954	−2.901, 2.910
	95% CI for B	0.000, 0.005	−0.180, 0.212	−8.672, −0.901	−8.678, 2.869
	t	2.182	0.161	−2.449	−0.998
	p	0.031	0.872	0.016	0.321
SRS total scores^a^	Beta (β)	0.171	0.011	−0.324	−0.113
	B, SE	0.000	0.002, 0.021	−1.283, 0.419	−0.696, 0.629
	95% CI for B	0.000, 0.001	−0.040, 0.044	−2.116, −0.450	−1.944, 0.552
	t	1.696	0.104	−3.063	−1.107
	p	0.093	0.917	0.003	0.271
Confirmatory dataset					
CBCL‐ADHD total scores^b^	Beta (β)	0.201	‐	‐	‐
	B, SE	60.417, 36.498			
	95% CI for B	0.000, 0.001	‐	‐	‐
	t	1.655			
	p	0.103			
SRS total scores^b^	Beta (β)	0.011	‐	‐	‐
	B, SE	4.208, 44.862			
	95% CI for B	−0.001, 0.001	‐	‐	‐
	t	0.094			
	p	0.926			

^a^ As reported in Table S4.1, consistent results were found when t‐scores were used rather than total scores.

^b^ Results of the regression analyses using t‐scores are reported in Table S4.2 for transparency, and are in a similar direction but of smaller magnitude.

A pre‐registered (after analysis of the exploratory dataset) regression using the exploratory and confirmatory datasets combined showed that ADHD traits were positively associated with Success Score (*F* (1,169) = 5.219, *β* = 0.173, *p* = 0.024, *r* ^2^ = 0.030).

#### Autism traits

**FIGURE 5:**
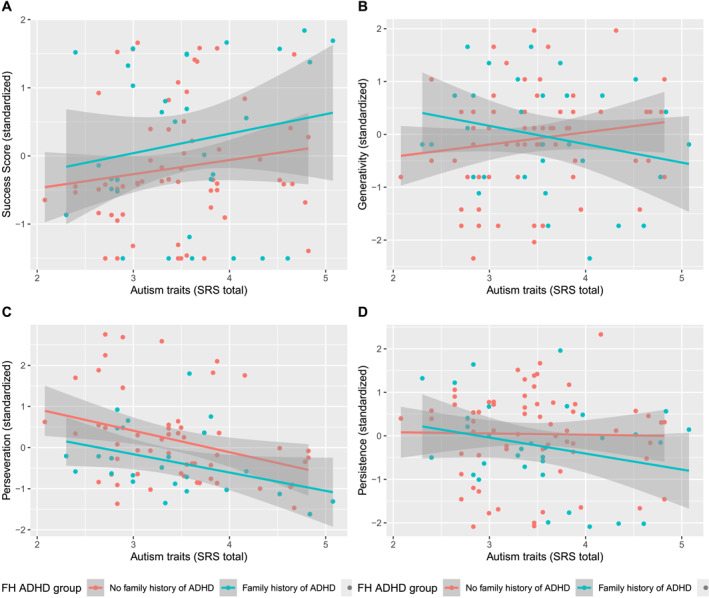
Regression of standardised proble‐solving variables (A) Success Score; (B) Generativity; (C) Perseveration; (D) Persistence) on autism traits (log transformed), by FH‐ADHD group in the exploratory dataset.

**FIGURE 6:**
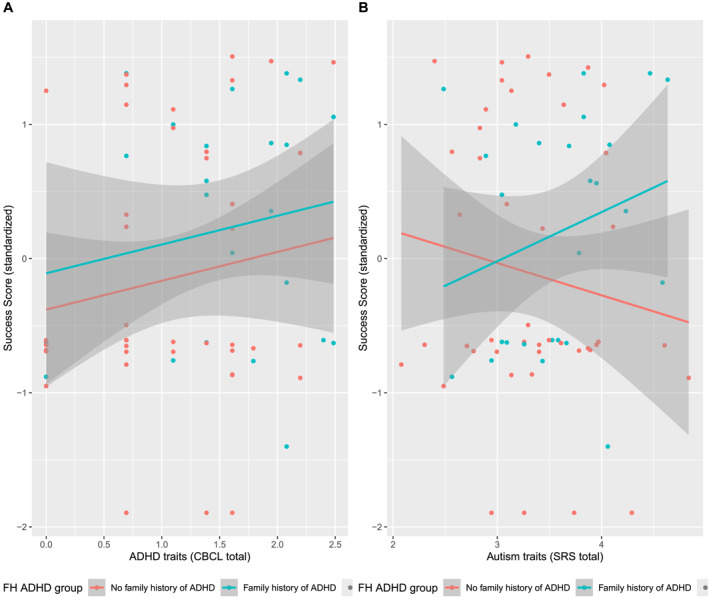
Regression of problem‐solving Success Score (standardised) on ADHD traits (log transformed) (A) and autism traits (log transformed) (B), by FH‐ADHD group in the confirmatory dataset.

As shown in Table 3, in the exploratory dataset, autism traits were negatively associated with Perseveration, and with a trend‐level positive association with Success Score; see also Figure 5. In the confirmatory dataset, the hypothesized positive autism trait‐Success Score association was not observed; see Figure 6.

In a pre‐registered (after analysis of the exploratory dataset) regression using the exploratory and confirmatory datasets combined, the hypothesized positive autism trait‐Success Score association was not observed (*F* (1,167) = 2.294, *β* = 0.117, *p* = 0.132,*r* ^2^ = 0.014).

## DISCUSSION

The first, exploratory, stage of this study revealed that UK 2‐ and 3‐year‐olds with a family history of ADHD show advantages in problem‐solving when completing a reward‐retrieval challenge (more rewards retrieved, faster, leading to higher Success Scores), relative to peers with no family history of ADHD). Moreover, parent‐reported ADHD traits at age 3 years were positively associated with higher Success Scores on our problem‐solving task. We partially replicated this in a confirmatory dataset of Swedish 3‐year‐olds, whereby children with family history of ADHD (and autism) showed a trend towards higher Success Scores‐. In the combined datasets, children with a family history of ADHD showed significantly higher Success Scores (with a small‐to‐medium effect) compared with peers, and ADHD traits were positively associated with problem‐solving scores, albeit accounting for only 3% of the variance.

This study is the first to demonstrate an ADHD‐related advantage for problem‐solving in children as young as 3 years, and is consistent with prior research with older children and adults indicating that ADHD may confer an advantage in generating multiple solutions to a problem (Boot et al., 2020; Girard‐Joyal & Gauthier, 2022; Hoogman et al., 2020; Stolte et al., 2022). Some researchers have suggested that these advantages may be limited to individuals with elevated but sub‐clinical ADHD traits (Hoogman et al., 2020). As shown in Figures 4 and 6, in our sample, high problem‐solving scores were observed for those with ADHD trait scores in the clinical range.

We did not find convincing evidence for autism‐related differences in problem solving. Previous studies have reported increased perseveration and reduced generativity amongst autistic children (Granader et al., 2014; Lai et al., 2017). In contrast, in our exploratory dataset, autism traits were associated with less perseveration, meaning that children with autistic traits tended to spend less of their overall manipulation time stuck on one strategy. However, in early childhood, perseveration on a response may be an adaptive stage on the path to skilled behaviour (Hendry et al., 2016) and the trend‐level positive association between autism traits and overall problem‐solving success amongst 3‐year‐olds in the exploratory dataset was not replicated in the confirmatory dataset. Therefore, we do not consider this compelling evidence for autistic strengths in problem‐solving. We also found no evidence that a family history of autism was associated with differences with problem‐solving in either direction.

In the confirmatory sample, the family history of ADHD advantage was only at trend level when participants coded as FH‐autism‐only were included in the comparison. When children coded as FH‐autism‐only were excluded (in exploratory follow‐up analyses) the contrast between children with and without a family history of ADHD was significant, with a large effect size. As noted in the introduction, family history of autism confers increased likelihood of ADHD, and vice versa (Ghirardi et al., 2018; Miller et al., 2019). Consistent with this, ADHD traits were elevated (and above clinical thresholds for some individuals) in the FH‐autism‐only groups of the exploratory and confirmatory sample, and highest in the FH‐autism‐and‐ADHD group of in the exploratory sample (we did not have an equivalent group in the confirmatory sample). Our data cannot speak precisely to how familial liability for autism and ADHD interact to shape phenotypic expression (and this may change over the course of development) but it is plausible that children who have a family history of autism and ADHD are most likely to show elevated ADHD traits and the associated cognitive phenotype at age 3, followed by children with a family history of ADHD only, followed by children with a family history of autism, with children with no family history of ADHD or autism being least likely to show elevated ADHD traits and associated cognitive profiles. This could account for the pattern of Success Scores observed in our study, whereby children who have a family history of autism and ADHD are most likely to achieve high Success Scores, followed by children with a family history of ADHD only, followed by children with a family history of autism.

As noted above, effect sizes relating to ADHD‐related differences in problem solving were modest. This may partly be due to the high measurement noise when considering ADHD traits at this young age (Curchack‐Lichtin et al., 2014; Nili et al., 2023), and to the generally modest levels of ADHD traits observed in our sample. Further, under versions of the Diagnostic and Statistical Manual of Mental Disorders prior to 2013 (APA, 2013), it was not possible to have a diagnosis of both autism and ADHD—even if the individuals showed strong traits of both. Consequently, there are likely to be children allocated to the ‘FH‐autism‐only’ group who also in fact have a first‐degree relative with (undiagnosed) ADHD. This may have particularly impacted the confirmatory sample where participants were coded as having a family history of ADHD only if a clinical diagnosis of ADHD was reported in the sibling or parent (as opposed to the exploratory sample where participants could also be coded as having a family history of ADHD if their parent or sibling scored above threshold on a clinical checklist; see Supporting Information S1: 1).

Many factors other than family history of ADHD and child ADHD traits also influence children's problem‐solving performance, likely including developmental ability, and variation in the home and wider early learning environment linked to socio‐economic factors. Our current results (which did not include covariates due to insufficient power), indicate that children with FH‐ADHD tend to show an advantage in problem‐solving scores even though, at a group level, they tend to have lower developmental ability and maternal education levels. Future research with larger samples should consider how child and environmental factors interact with family history status in order to illuminate potential mechanisms of transmission, and to build a better understanding of the conditions under which children are likely to develop strong problem‐solving skills.

### Explanations for an ADHD advantage in problem‐solving in early childhood

Why might the ADHD advantage in performance on this problem‐solving task occur? One clue comes from work showing that in a general population sample of 1.5‐ to 4‐year‐olds who completed the Problem‐Solving Box task, low inhibitory control is linked to greater overall problem‐solving success, greater generativity, and lower perseveration (Hendry et al., 2022). Similarly, Vaisarova and Carlson (2021) have shown that 4‐ to 6‐year‐olds’ performance on a divergent thinking task was negatively associated with their performance on a battery of executive function tasks and positively associated with individual differences in surgency (a dimension of temperament indexing children's activity level, sociability, and enjoyment of highly stimulating activity which has been previously linked to ADHD (Nigg, 2022; Shephard et al., 2022)). Meanwhile, Chrysikou (2019) has summarised research that indicates that lower prefrontal cortex regulation may lead to the relaxation of top‐down inhibitory constraints, which in turn creates beneficial conditions for divergent thinking (i.e. generativity of novel ideas or potential solutions to a problem). Meta‐analyses of brain imaging studies have found that ADHD participants show less activity in prefrontal cortex (amongst other areas) when engaged in tasks requiring inhibitory control, compared with controls (Cortese et al., 2012; McCarthy et al., 2014). Similarly children with a family history of ADHD who did not meet diagnostic criteria for ADHD themselves, show reduced activity in parts of the prefrontal cortex compared to controls when engaged in a task requiring inhibitory control (Durston et al., 2006; Mulder et al., 2008; van Rooij et al., 2015). Thus, the patterns of brain activation that cause individuals with a diagnosis or family history of ADHD to struggle with classic measures of inhibitory control may potentially be the same patterns that enable those same individuals to do well on a problem‐solving task requiring generativity of potential solutions.

An alternative possibility is that children in the FH‐ADHD group, and those with high ADHD traits, had a stronger response to the extrinsic motivation of the task (i.e. were willing to work harder to retrieve the treats). Researchers have shown that children with ADHD are more active when motivated by competition and extrinsic reward (Skalski et al., 2021) and that adults with ADHD became more generative under conditions of extrinsic motivation (Boot et al., 2020). Of note though, this literature points towards increasing motivation as a way of levelling the field for individuals with ADHD, rather than suggesting that motivation alone can produce an ADHD advantage. Indeed, our results contrast with a wider literature linking ADHD to lower performance on ‘hot EF’ tasks (i.e. requiring goal‐directed behaviour in motivationally or emotionally salient situations) (Smith et al., 2024). To date, this literature has largely centred on school‐aged children and adolescents with ADHD; this study highlights the need for further investigation into both developmental changes in ADHD‐related differences in hot EF, and the conditions under which these differences manifest as strengths versus difficulties. Relatedly, future studies in which the salience of the rewards is manipulated might provide useful insight into the conditions under which children with high ADHD and/or autistic traits show high or low levels of perseveration and generativity.

### Strengths and limitations of the study

The study's family history design enabled us to consider the potentially contradictory effects of family history of autism and ADHD on problem solving—although this could have been further enhanced by the inclusion of a FH‐ADHD‐only (i.e. no‐FH‐autism) group in the confirmatory sample. It does however raise the possibility of environmental effects as an explanation for the FH‐ADHD advantage: Children used to observing family members tackle problems in a highly generative way may be more likely to take such an approach themselves. Alternatively, we speculate that children with a FH‐ADHD might be more likely to be raised in environments that are not highly predictable (Agnew‐Blais et al., 2022) and/or where parents do not always immediately respond to all bids for assistance (because they are attending to the needs of other children, are otherwise distracted themselves, or simply value child autonomy); over time children with a FH‐ADHD might learn from these cues to adopt a more independent, proactive problem‐solving style (Ellis et al., 2017). This does not undercut our conclusions, but rather opens up a further potential explanation for the advantages we observed.

Our sample size was smaller than anticipated for the confirmatory sample, and therefore under‐powered to detect an effect of family history of ADHD of the size observed in the exploratory sample (with 65% power to detect a medium effect). However, the consistency of the direction of effects across samples (which, when combined, yielded 90% power to detect the observed effect size, using a one‐tailed *t*‐test), and fact that every child in the confirmatory sample with a family history of ADHD retrieved at least 1 reward, increases our confidence in our conclusion that problem‐solving (on an ill‐structured, highly motivating task) is a strength for toddlers with a family history of ADHD, and those with high ADHD traits. Similarly, although the confirmatory regression analyses were under‐powered (with only 50% power to detect a significant association between ADHD traits and problem‐solving scores of the effect size observed in the exploratory dataset), confirmatory associations were of a similar magnitude and direction to the exploratory results, hence were significant when the two samples were combined.

### Clinical implications

Our research does not set out to underplay cognitive difficulties associated with ADHD or autism, but rather to complement research into areas of difficulties by adding to the evidence‐base on less well‐studied but equally important aspects of cognition. In this way, we hope to build a more accurate and well‐rounded understanding of neurodivergent cognitive profiles. In turn this approach may help to reduce stigmatising and inaccurate perceptions of neurodivergence as being a negative phenomenon, and thus indirectly contribute to improving neurodivergent children's self‐esteem and long‐term mental wellbeing.

Clinicians, educationalists and Special Educational Needs professionals are already aware of the need to offer individualised support to children based on their specific profile of strengths and difficulties. Our study shows that considering a child's family history as well as their neurodivergent traits may help identify where those strengths are likely to lie: Specifically, we show that children with ADHD traits and/or a family history of ADHD (with and without family history of autism) are likely to have an aptitude for generative problem‐solving. Embedding skills practice or puzzles in scenarios where children are playfully challenged to identify alternative solutions may thus be one way to build new skills whilst consolidating strengths. Such an approach has been used in the Supporting Toddlers with a connection to autism or ADHD to develop strong Attention, Regulation and Thinking skills (START) programme, which has been found to be acceptable for families of toddlers with a family history of autism and/or ADHD (Hulks et al., 2024). Further, we hope that our approach of using a highly motivating, ecologically valid task with low demands in domains that might still be challenging (such as language and motor control, planning, or compliance with an arbitrary rule) provides a useful example of how the learning or assessment environment can be structured to enable children to best show their capabilities.

## AUTHOR CONTRIBUTIONS

**Alexandra Hendry:** Conceptualization; data curation; formal analysis; investigation; methodology; writing ‐ original draft; writing ‐ review and editing. **Emily J. H. Jones:** Funding acquisition; methodology; supervision; writing ‐ review and editing. **Linn Andersson‐Konke:** Investigation; project administration; writing ‐ review and editing **Mary Agyapong:** Investigation; methodology; writing ‐ review and editing. **Tessel Bazelmans:** Investigation; writing ‐ review and editing. **Jannath Begum‐Ali:** Data curation; project administration; writing ‐ review and editing. **Mutluhan Ersoy:** Investigation; writing ‐ review and editing. **Amy Goodwin:** Data curation; project administration; writing ‐ review and editing. **Greg Pasco:** Data curation; investigation; writing ‐ review and editing. **Terje Falck‐Ytter:** Conceptualization; funding acquisition; resources; writing ‐ review and editing. **Mark H. Johnson:** Funding acquisition; resources; writing ‐ review and editing. **Tony Charman:** Funding acquisition; supervision; resources; writing ‐ review and editing.

## CONFLICT OF INTEREST STATEMENT

TC has served as a paid consultant to F. Hoffmann‐La Roche Ltd. and Servier; and has received royalties from Sage Publications and Guilford Publications. EJ is a Joint Editor on JCPP Advances. MJ receives royalties from Wiley‐Blackwell, OUP and MIT Press. GP is a licenced ADOS‐2 trainer and receives income from delivering ADOS‐2 training.

## ETHICAL CONSIDERATIONS

In all cases, parents provided informed consent and the study was performed in accordance with relevant ethical guidelines and regulations. Ethical approval was granted by the National Research Ethics Service London Research Ethics Committee (13/LO/0751) and from the Birkbeck Psychological Sciences Ethics Committee (121,373, 13/1516) (exploratory dataset) and by the local ethics committee in Uppsala Sweden (EPN; Dnr. 2013/241) (confirmatory dataset).

## Supporting information

Supporting Information S1

## Data Availability

Data available following a review of requests as indicated here: https://www.basisnetwork.org/collaboration‐and‐project‐affiliation.
