# Supplementary material for: Family history of ADHD associates with stronger problem‐solving skills amongst 2‐ to 3‐year‐olds
Source: JCPP Adv. 2025 Mar 12;5(4):e70009. doi: 10.1002/jcv2.70009 (PMC12698283; doi:10.1002/jcv2.70009)
Supplement: Supplementary file 1 — Supporting Information S1 [file JCV2-5-e70009-s001.docx]

Supporting Information 1: Further detail on participants

# Participants: exploratory dataset

Participants were recruited for a longitudinal study running from 2013 to 2019 from a volunteer database, community flyers, internet adverts and clinical networks. Inclusion criteria for all participants included full-term birth (gestational age greater than 36 weeks) and an older sibling. At the time of enrolment, none of the participants had a known medical or developmental condition.

Before families enrolled in the study, a telephone screening form was used to determine the presence of autism and ADHD in family members. Where possible, families who reported suspected ADHD at study entry were screened using a shortened version of the Conners. For siblings (aged less than 6 years), a shortened version of the Conners Early Childhood (Conners, 2008, Conners & Goldstein, 2009) form was used. For siblings (6 years or older), a shortened version of the Conners 3 was used. Thresholds for inclusion were the presence of 6 ADHD traits on either the hyperactivity/impulsivity or inattention scale, and a positive score on the impairment scale. For parents a shortened version of the Conners Adults ADHD Rating Scale (CAARS; Conners et al., 1999) was used. Thresholds for inclusion were the presence of 5 ADHD traits on either the hyperactivity/impulsivity or inattention scale as per DSM-5 guidelines.

# Participants: confirmatory dataset

Participants were recruited for a longitudinal study running from 2011 to 2021. Inclusion criteria included full-term birth (gestational age greater than 36 weeks) and an older sibling. At the time of enrolment, none of the participants had a known medical or developmental condition.

Problem-solving performance data from the No-FH-autism/ADHD’ groups of both datasets has been previously reported in BLINDED FOR REVIEW but performance data from the FH-autism, FH-ADHD and FH-autism-and-ADHD groups has not previously been reported. Questionnaire data has been previously reported in BLINDED FOR REVIEW.

In both datasets, participant families were reimbursed expenses for travel, subsistence and overnight stay if required. In the exploratory dataset, participants were also given a certificate and t-shirt after each visit.

*Table S1.1* Reasons for missing Problem-Solving Box data, by FH group

| Reasons for missing/  incomplete data | No FH-autism/ADHD | FH-autism only | FH-autism+ADHD | FH-ADHD only |  |
| --- | --- | --- | --- | --- | --- |
| Exploratory dataset |  |  |  |  |  |
| *2-year visit* | | | | | |
| Task not attempted (remote assessment, out of time, or only questionnaire data collected) |  | 3 | 0 | 1 |  |
| Child would not attempt, or could/would not do after demo |  | 3 |  |  |  |
| Admin error |  | 3 |  |  |  |
| Technical issue |  | 6 | 1 |  |  |
| Only success score available due to file corruption |  | 2 | 2 | 1 |  |
| Success score not computed as task incorrectly terminated after 3 minutes | 1 | 1 |  |  |  |
| *3-year visit* | | | | | |
| Task not attempted (remote assessment, out of time, or only questionnaire data collected) | 4 | 5 | 0 | 1 |  |
| Child would not attempt, or could/would not do after demo | 0 | 1 | 0 | 0 |  |
| Admin error | 0 | 1 | 0 | 0 |  |
| Technical issue | 0 | 4 | 0 | 0 |  |
| Confirmatory dataset | **No FH-autism/ADHD** | **FH-autism only** | **FH-autism+ADHD** | **FH-ADHD only** |  |
| Child quit before 2 minutes | 0 | 1 | 0 | N/A |  |

Supporting Information 2: Coding

# Coding

Recordings were produced at a frame rate of 25fps and coded with Mangold Interact v16. Data were coded by 1 lead coder (MA) and 5 additional student coders AO, BC, FH, IG and MH. Student coders were trained by either the lead author or lead coder and only contributed data after demonstrating high inter-rater reliability (Intraclass correlation coefficient calculated using 2-way random effects average measures of .8 or higher) on each of the variables.

# Coding scheme

Videos were coded according to the coding scheme indicated below where each code indicates a continuous, mutually-exclusive behaviour. Behaviours that were either defined a priori as goal-directed manipulation, or observed during coding and subsequently agreed by the coding team to be goal-directed are indicated in italics. Behaviours considered as non-goal-directed sensory-motor behaviours are indicated in bold. Note that a given strategy (e.g. lid lifting) was considered distinct in each location to which it was applied (e.g. left-hand, central and right-hand lids).

Table S2.1 Behavioural codes for the Problem-Solving Box Task

| Code | Notes |
| --- | --- |
| *push down left lid* | Child presses down on either the green knob or the compartment itself |
| *push down centre* | Child presses down on either the green knob or the compartment itself |
| *push down right* | Child presses down on either the green knob or the compartment itself |
| *peeling up (anywhere)* | Child uses nails/fingertips to exert pressure on a small part of the box, generally near to a join in materials, seemingly to peel off a layer |
| *squeezing/pinching (anywhere)* | Child uses fingertips and/or thumbs to exert pressure on a small part of the box, seemingly to interrogate its physical properties |
| *lift lid – left* | Child pulls on the green knob on the left of the box. This action will not be successful so is coded any time the child applies some force (gripping/pulling) |
| *lift lid – centre* | Child pulls on the green knob in the centre of the box (the lid will lift when pulled) |
| *lift lid – right* | Child pulls on the green knob on the right of the box. This action will not be successful so is coded any time the child applies some force (gripping/pulling) |
| *pull string – left* | Child touches and exerts force on the string attached to the drawer on the left side of the box. This action will not be successful so is coded any time the child applies some force. Just fiddling with the string is coded as touching box but no obvious manipulation. |
| *pull string – right* | Child touches and exerts force on the string attached to the drawer on the right side of the box. This action will not be successful so is coded any time the child applies some force. Just fiddling with the string is coded as touching box but no obvious manipulation. |
| *pull ribbon – left* | Child touches and exerts force on the ribbon. If child exerts force but doesn’t manage to move the drawer (i.e. because pulling from an angle) still code as a pull. |
| *pull ribbon – right* | Child touches and exerts force on the ribbon. If child exerts force but doesn’t manage to move the drawer (i.e. because pulling from an angle) still code as a pull. |
| *push drawer – left* | Child pushes on drawer (rather than pulling string/ribbon) |
| *push drawer – centre* | Child pushes on drawer (rather than pulling string/ribbon) |
| *push drawer – right* | Child pushes on drawer (rather than pulling string/ribbon) |
| *push whole unit* | Child uses arms, legs or whole body to move whole unit away from their body |
| *lift whole unit* | Child uses arms to lift unit (may achieve only a small lift) |
| *pull whole unit* | Child uses arms, legs or whole body to move whole unit away towards their body |
| *pressing down on unit* | Child uses hands, arms or body to push down on the unit (if the pressing down directly precedes a climbing action, code as part of climbing) |
| *trying to access through central compartment* | Child places fingers/hands against the wall of the central compartment adjacent to the other compartments. Note that this behaviour looks different to reaching back into the central compartment as they are exploring the boundaries of the compartment |
| *peeling up with tool (anywhere)* | Child uses an object (e.g. the central lid) to try to lever off the fixed lids. This behaviour is different from using an object to bang the box (which is coded under “tapping or banging”): the angle and pressure applied indicates that the child is trying to force *up* the top layer of the box. |
| *push side left* | Child pushes on fixed side of the box (left hand side) |
| *push side right* | Child pushes on fixed side of the box (right hand side) |
| **sitting / climbing on unit** | Child climbs or sits on unit |
| **crawling through unit** | Child crawls through table to which the box is fixed |
| **tapping or banging** | Child uses hands or another object/part of the box to bang/tap the box/unit |
| **licking** | Child licks the box (anywhere) |
| not touching* | Child’s fingers/hands/body are not touching box (accidental brushing of the box whilst moving past can be coded as not touching). |
| touching box but no obvious manipulation* | Child’s fingers/hands/body rest on the box (or attached table) but no pressure is applied and no other strategy is evident. |
| replacing lid* | Child replaces central compartment lid |
| replacing drawer* | Child pushes back drawer into position |
| replacing reward* | Child places reward back into the compartment after retrieval |
| re-retrieving reward* | Retrieving reward after it had already been retrieved at least once |
| strategy obscured* | Use this code only where strategy is entirely obscured. |

*these behaviours are included in overall task duration calculations but are not considered as goal-directed strategies, nor as sensory-motor exploration.

Supporting Information 3: Pre-registration

The following analysis plan was registered 27 October 2022, following analysis of the exploratory dataset, but prior to accessing and analysing the confirmatory dataset. The date-stamped registration is available from [BLINDED FOR PEER REVIEW].

## Hypotheses

1. Three-year-olds with a family history of ADHD will have higher Success Scores on the Problem-Solving Box task compared with peers with no family history of ADHD.

2. At age 3 years, ADHD traits will be positively associated with Success Scores on the Problem-Solving Box task.

3. At age 3 years, autism traits will be positively associated with Success Scores on the Problem-Solving Box task.

# Analysis Plan

To test Hypothesis 1 we will use a one-tailed independent sample t-test with Success Score as the outcome variable, and Family History of ADHD as the predictor variable. If age is found to differ significantly between groups, an ANCOVA will also be run with Success Score as the dependent variable, Family History of ADHD as the fixed factor, and age as a covariate.

To test Hypothesis 2 we will use a linear regression with Success Score as the dependent variable, and CBCL-ADHD scale total t-score as the independent variable.

To test Hypothesis 3 we will use a linear regression with Success Score as the dependent variable, SRS total t-score as the independent variable.

# Inference criteria

The criterion for significance is set to p<.05. 95% confidence intervals will also be reported for all tests. All tests will be reported.

# Data exclusion

Outliers will be included in the analyses. Data will only be excluded if the behavioural task (Problem-Solving Box task) was judged by the administrator or coder to be invalid (due to administration or technical error, or child refusal) or if missing, as described below.

# Missing data

Participants with incomplete data will be included in those analyses for which data are available, but excluded from those analysis for which data are not available: e.g. a participant with valid Problem-Solving Box Success Scores, Family History of ADHD data but no clinical trait measures will be included in tests for Hypothesis 1 but not 2 or 3.

# Exploratory analysis

To evaluate whether consistent findings are achieved in a larger dataset, and whether trend-level associations reach significance thresholds when the test has higher power, we will re-run the tests described above with the exploratory and pre-registered datasets combined.

Supporting Information 4: Trait associations using t-scores

*Table S4.1*. Associations between PS box performance and autism/ADHD traits (t-scores) amongst 3-year-olds computed using linear regression of PS box performance on autism/ADHD traits; exploratory dataset.

|  |  | Success Score | Generativity | Perseveration | Persistence |
| --- | --- | --- | --- | --- | --- |
| CBCL-ADHD  t-scores | Beta | .211 | .008 | -.285 | -.086 |
|  | B, SE | 8.135, 3.751 | .004, .050 | -.008, .003 | -.001, .002 |
|  | 95% CI for B | 0.000, 0.010 | -0.378,0.408 | -18.269, -2.952 | -16.618, 6.536 |
|  | t | 2.169 | .076 | -2.754 | -.864 |
|  | p | .032 | .939 | .007 | .390 |
| SRS  t-scores | Beta | .174 | -.004 | -.335 | -.090 |
|  | B, SE | 3.597, 2.084 | -.001, .027 | -.005, .001 | -.001, .001 |
|  | 95% CI for B | -0.001, 0.018 | -0.783, 0.749 | -39.188,-9.026 | -32.996,12.665 |
|  | t | 1.726 | -.044 | -3.181 | -.884 |
|  | p | .088 | .965 | .002 | .379 |

*Table S4.2.* Associations between PS box performance and autism/ADHD traits (t-scores) amongst 3-year-olds computed using linear regression of PS box performance on autism/ADHD traits; confirmatory dataset.

|  |  | Success Score |
| --- | --- | --- |
| CBCL-ADHD t-score | Beta | .151 |
|  | B, SE | .004, .003 |
|  | 95% CI for B | -0.002, 0.009 |
|  | t | 1.228 |
|  | p | .224 |
| SRS t-score | Beta | -.001 |
|  | B, SE | -.000, .005 |
|  | 95% CI for B | -0.010, 0.010 |
|  | t | -.006 |
|  | p | .996 |

Supporting Information 5: Associations between Problem-Solving Box variables

Consistent with previous research (Hendry et al., 2022), at both the 2- and 3-year visits Success Score is positively correlated with in-task indices of generativity and persistence, and negatively correlated with perseveration; Supplementary Tables 5.1 and 5.2.

*Table S5.1.* Pearson correlations between Success Score variables in the exploratory dataset at the 2-year visit

|  | Success score | Generativity | Perseveration |
| --- | --- | --- | --- |
| Generativity | .624*** |  |  |
| Perseveration | -.425*** | -.772*** |  |
| Persistence | .669*** | .677*** | -.383** |

***p<.001, **p<.05, *p<.01

*Table S5.2.* Pearson correlations between Success Score variables in the exploratory dataset at the 3-year visit

|  | Success score | Generativity | Perseveration |
| --- | --- | --- | --- |
| Generativity | .454*** |  |  |
| Perseveration | -.493*** | -.502*** |  |
| Persistence | .324*** | .585*** | .082 |

***p<.001, **p<.05, *p<.01
